# Plasma omega-3 PUFA and white matter mediated executive decline in older adults

**DOI:** 10.3389/fnagi.2013.00092

**Published:** 2013-12-16

**Authors:** Gene L. Bowman, Hiroko H. Dodge, Nora Mattek, Aron K. Barbey, Lisa C. Silbert, Lynne Shinto, Diane B. Howieson, Jeffrey A. Kaye, Joseph F. Quinn

**Affiliations:** ^1^Brain Institute, Department of Neurology, Oregon Health and Science UniversityPortland, OR, USA; ^2^Beckman Institute for Advanced Science and TechnologyUrbana, IL, USA

**Keywords:** omega-3 fatty acids, white matter hyperintensity, cognitive decline, memory, hypertension

## Abstract

**Introduction:** Cross-sectional studies have identified long chain omega-3 polyunsaturated fatty acids (eicosapentaenoic acid 20:5n-3 and docosahexaenoic acid 22:6n-3 (O3PUFA) in association with fewer white matter lesions and better executive function in older adults. We hypothesized that O3PUFA are associated with less executive decline over time and that total white matter hyperintensity volume (WMH) mediates this association.

**Methods:** Eighty-six non-demented older adults were followed over 4 years after measurement of plasma O3PUFA with annual evaluations of cognitive function. A subset of these participants also had brain MRI of total WMH available to conduct a formal mediation analysis of a putative relationship between O3PUFA and cognitive function.

**Results:** Mean age at baseline was 86, 62% were female and 11% carried the *APOE4* allele. Each 100 μg/ml increase in plasma O3PUFA associated with 4 s less change in executive decline per year of aging (*p* = 0.02, fully adjusted model). O3PUFA was not associated with verbal memory or global cognitive changes. The significance of the association between O3PUFA and better executive function was lost once WMH was added to the regression model.

**Conclusion:** Executive decline with age appears to be a cognitive domain particularly sensitive to plasma O3PUFA in longitudinal examination. O3PUFA may modulate executive functioning by mechanisms underlying the development of WMH, a biologically plausible hypothesis that warrants further investigation.

## Introduction

Delaying the onset of Alzheimer's disease and other late life dementias is an agenda budding across the globe. Characterizing the heterogeneity in structural and functional brain changes that relate to dementia risk will enable preventive therapies to target these characteristics in populations primed to gain benefit. Diet and nutrition pose a significant opportunity for prevention; however, success here also requires knowledge of the risk profile in order to engage appropriately.

Cerebral white matter hyperintensities (WMH), largely considered a marker of small vessel disease and dementia risk, are of significant interest to prevention strategies (Schmidt et al., 2003; Garde et al., 2005; Bastos Leite et al., 2006; Kramer et al., 2007; Silbert et al., 2008; Debette et al., 2010; Gorelick et al., 2011). These WMH are prevalent in 60–92% of non-demented elders age 65 and older and increase risk for cognitive impairment (de Leeuw et al., 2001; Silbert et al., 2009). WMH represents an early structural MRI risk factor for Mild Cognitive Impairment and dementia (Silbert et al., 2012), and thus, provides an attractive therapeutic target. The design of rational therapy for this early brain structural change requires knowledge of its pathophysiology. Both intake (Virtanen et al., 2008) and peripheral concentration of long chain omega-3 polyunsaturated fatty acids (O3PUFA) are associated with less WMH in cross–sectional studies (Bowman et al., 2012; Tan et al., 2012; Virtanen et al., 2013). Although the cognitive consequences associated with WMH accumulation may ultimately pervade several domains, executive dysfunction with impaired information processing and cognitive flexibility have been noted as early indicators of its accrual (Schmidt et al., 1993; DeCarli et al., 1995; Adak et al., 2004; Brickman et al., 2006; Verdelho et al., 2007). Intrigued by this fabric of literature, we tested the hypothesis that O3PUFA associates with less executive decline, and that WMH mediates the relationship between these fatty acids and executive function.

## Materials and methods

### Study population

The Oregon Brain Aging Study (OBAS) is a cohort study of brain aging in people age 65 and older free of usual confounding factors known at the time to modify the risk for cognitive decline (i.e., vascular disease, smoking, stroke, diabetes) (Kaye et al., 1994). Enrollment was opened in 2004 to also include volunteers with stable chronic conditions common with advanced age (i.e., hypertension, diabetes) to better represent the general population. Clinical, neuropsychometric and brain MRI are collected annually. Clinical Dementia Rating (CDR) (Morris, 1993) scale is based on interviews with the participant and their collateral historian about functioning and cognitive skills in conjunction with the MMSE (Folstein et al., 1975) and the Cognistat (Kiernan et al., 1987). Blood was collected appropriately for nutrient biomarkers beginning in the 2006–2007. Inclusion was restricted to non-demented participants (CDR ≤ 0.5). Eighty-six participants had plasma fatty acids and psychometric measures available for longitudinal analysis. Thirty-two of these 86 also had MRI collected at the time of the nutrient blood draw to permit a formal mediation analysis.

### Standard protocol approval and patient consent

Informed consent was obtained from all subjects for participation in this study, which was approved by the institutional review board for human study at Oregon Health & Science University.

### Biomarker acquisition and analysis

#### Plasma long-chain omega-3 fatty acids

Fasting blood was collected between the hours of 0700 and 1200 noon Pacific Standard Time beginning in September of 2006 and ending December 2007. Total lipid long chain n-3 polyunsaturated fatty acids as methyl esters were quantified using gas chromatography equipped with flame ionization detector and expressed as absolute plasma concentrations (μ g/mL)(Bowman et al., 2012). Plasma eicosapentaenoic acid (20:5n-3) and docosahexaenoic acid (22:6n-3) were combined (O3PUFA).

#### Volumetric brain MRI

Brain regions of interest were obtained using MRI 1.5 T magnet and REGION image analysis software. The procedures have been previously described (Mueller et al., 1998). Briefly, the sums of pixel areas for all slices were converted to volumetric measures by multiplying by the slice thickness for each of the following regions of interest: total white matter hyperintensity volume (WMH includes periventricular and subcortical deep signals) and supratentorial brain volume as total cerebral brain volume (TBV, excluding cerebellum and brain stem). Regression for brain tissue, CSF, and WMH collectively against bone creates a boundary along the inner table of the skull to determine the total intracranial volume. Additional boundaries were manually traced along the tentorium cerebelli and the superior border of the superior colliculus, the pons, and the fourth ventricle. The pituitary, vessels in the sphenoid region, and any sinuses that may have been included by the automatic regression were excluded manually. Using REGION's sampling tools, the analyst selects representative, unambiguous pixels of WMH (as well as brain tissue, fluid, and bone) from the multi-echo sequence display. Proton and T2 intensities are included in a regression model taking into account the location of each pixel that differentiates the tissue types. Distinction of WMH from brain tissue and fluid is achieved by visualizing higher signal intensities on proton density and T2 images. Inter-rater reliability coefficients using this approach for white matter segmentation are 0.85 and >0.95 for total intracranial, brain, and ventricular regions of interest.

### Primary outcomes and potential confounders

#### Neuropsychometrics

Previous cross-sectional analysis (Bowman et al., 2012) demonstrated O3PUFA in association with Trail Making Test Part B (Reitan, 1958), a measure commonly utilized to reflect executive function. This test was therefore used as our primary outcome measure for the current longitudinal analysis. WMS-R Logical Memory Story delayed (Wechsler, 1981) and the MMSE were also analyzed to resolve cognitive measures with apparent sensitivity to O3PUFA over time.

#### Potential confounders and other covariates

We utilized a parsimonious approach to our model building by including potential confounders on the basis of their previous association with cognitive decline. These included age (continuous), gender (man/women), education (continuous, years), *APOE* genotype determined using PCR (e4 carrier, y/n), hypertension (y/n), and depression (y/n). We restricted our covariates entered into the mediation analysis on the basis of their significant association with our outcomes of interest to avoid depleting degrees of freedom in a limited sample size (Supplementary Material, age and *APOE4* carrier status, total intracranial volume). Covariates were collected and confirmed during the clinical interview (i.e., hypertension, depression).

### Statistical analysis

All statistics were performed in STATA v10.1 software (College Station, TX). Baseline differences in characteristics between those with and without MRI were calculated using independent t-test or Wilcoxon rank sum test for continuous variables and Pearson's chi-square test or Fisher's exact test for categorical variables as appropriate.

#### Longitudinal analysis

Linear mixed effects models estimated the mean and within-person slope of cognitive change by baseline plasma O3PUFA concentration. The mixed effects model accounts for the within-person correlations on repeated measures. The interaction term (O3PUFA x Age) represents the “effects” of the baseline O3PUFA on cognitive change over time (using age at visit as the time variable). We interpret this as the annual cognitive change per unit increase in baseline O3PUFA. In addition to considering O3PUFA as a continuous measure, we also examined the difference between O3PUFA 1-SD above the mean (>100 μg/ml) versus a lesser value in relation to cognitive change. O3PUFA dichotomized (>100 μg/ml or less) in this setting represents the between-group annual difference in slope of cognitive change (high vs. low).

#### Formal mediation analysis

This procedure was utilized in an attempt to better describe the role of O3PUFA in relation to WMH and cognitive function. Differences between participants with and without MRI are presented in Table 1. Our mediation analysis used the baseline data and a 3-step framework (Baron and Kenny, 1986). The first step attempts to reproduce the initial model that demonstrates the association between O3PUFA and cognitive function. In the second step, we examine the association between the proposed “mediator” (i.e., WMH) and cognitive function and O3PUFA with the mediator WMH. The third step includes O3PUFA and WMH as simultaneous predictors of cognitive function. Attenuation of the beta-coefficient >10% or loss of statistical significance (alpha level >0.05) was stated *a priori* to imply a mediation effect in this construct. These regression models were adjusted for variables that demonstrated a significant association with cognitive function in the study sample (alpha value <0.05, two-sided) (Supplementary Material).

**Table 1:** **Baseline characteristics of the study popualtion^a^**.

	*****n*** = **86****	**MRI available**		***P* for difference^b^**
		**No (*n* = 54)**	**Yes (*n* = 32)**	
Age, y	85.7 (10)	81.7 (11.0)	92.4 (3.5)	<0.0001
Female, *n* (%)	53 (62)	28 (52)	25 (78)	0.02
Years of education	15 (3)	15.3 (2.6)	14.5 (2.4)	0.12
*APOE4* carrier, *n* (%)	9 (11)	8 (15)	1 (3)	0.14
Hypertension, *n* (%)	36 (42)	20 (38)	16 (50)	0.27
Depression, *n* (%)	15 (17)	10 (19)	5 (16)	0.73
CDR of 0, *n* (%)	60 (70)	38 (70)	22 (69)	0.87
**PLASMA NUTRIENTS**				
EPA (20:5n-3), μ g/ml	16.5 (10.5)	16.9 (12.4)	15.8 (7.8)	0.66
DHA (22:6n-3), μ g/ml	68.1 (17.8)	67.1 (17.9)	69.6 (17.6)	0.48
O3PUFA (EPA+DHA)	84.5 (26.7)	84.1 (28.1)	85.4 (24.6)	0.61
Vitamin B12, pg/ml	659 (312)	666 (306)	646 (328)	0.73
**PSYCHOMETRICS**				
MMSE	28 (2)	28.2 (1.9)	27.5 (2.2)	0.17
Trails B	137 (77)	123 (73)	160 (79)	0.01
Paragraph recall	14.0 (4.7)	15.0 (3.8)	12.3 (5.6)	0.02
**VOLUMETRIC MRI, cc**				
Intracranial	–	–	1107.5 (106.4)	–
Brain	–	–	821.1 (84.7)	–
Ventricular	–	–	55.2 (16.5)	–
WMH	–	–	13.8 (8.8)	–

^a^Mean and standard deviation unless otherwise stated.

^b^Baseline differences between those with and without MRI calculated by independent t-test or Wilcoxon rank sum test for continuous variables and Pearson's chi-square or Fisher's exact test for categorical variables as appropriate.

APOE4, Apolipoprotein E epsilon 4 allele carrier; EPA, eicosapentaenoic acid; DHA, docosahexaenoic acid; Trails B, Trail Making Test – Part B; Paragraph recall, Wechsler memory scale revised; WMH, total white matter hyperintensity.

## Results

Sixty-two percent of the participants were female and the mean age at baseline was 86. Prevalence of *APOE4* allele carrier status was 11% (Table 1). The mean MMSE was 28. Forty-two percent were being treated for hypertension and 17% for depression. Vitamin B12 deficiency was prevalent in 4%. Mean duration of follow-up was 3.9 years (range 1–5) (Table 1). Annual change in Trail Making Test Part B (Trails B) as measured by speed in test performance was 3.5 (± 0.42) s, −0.2 (± 0.04) points on WMS-R Delayed Paragraph Recall, and −0.1 (±0.02) points on MMSE.

In the results that follow, all subjects are included in the evaluation of relationships between O3PUFA and cognitive change (*n* = 86), while only those with MRI are included in the mediation analysis (*n* = 32). The participants with MRI were older and predominantly female in comparison to those without MRI (Table 1).

### Plasma O3PUFA and cognitive decline (table 2)

Plasma O3PUFA (EPA+DHA) was associated with less executive decline (Trails B) after adjustment for age, gender, education, *APOE4*, hypertension, and depression (*p* = 0.02) (Table 2). The magnitude of the association can be interpreted as a 4 s less decline on Trails B per year for each 100 μg/ml increase in plasma O3PUFA. These estimates indicate that an individual with an O3PUFA concentration of 200 μg/ml might be expected to complete the Trails B task 4 s faster than an individual with an O3PUFA of 100 μg/ml per year of aging. Given that each year of aging in our population was associated with a mean increase in Trails B completion time of approximately 4 s over the duration of follow up, these estimates indicate a 1 year delay in age-dependent executive decline per 100 μg/ml baseline O3PUFA concentration. By contrast, decline in Paragraph Recall (*p* = 0.83) and MMSE (*p* = 0.21) had no apparent relationship with O3PUFA.

**Table 2:** **Plasma O3PUFA and cognitive decline in older adults over 4-years (*n* = 86)^a^**.

	**Trail B**			**Paragraph recall**			**MMSE**		
	**β**	**SE**	***P***	**β**	**SE**	***P***	**β**	**SE**	***P***
O3PUFA × Age	−0.04	0.02	0.02	0.00	0.00	0.83	0.00	0.00	0.21
O3PUFA baseline	−0.66	0.27	0.02	0.01	0.02	0.56	0.01	0.01	0.19
Age	6.66	1.53	0.00	−0.19	0.11	0.10	−0.19	0.05	0.00
Gender	14.61	10.81	0.18	1.56	0.83	0.06	−0.05	0.22	0.81
Education	−1.37	2.74	0.62	0.17	0.18	0.36	−0.01	0.07	0.88
*APOE4*	15.19	13.99	0.28	0.09	1.17	0.94	−0.34	0.31	0.28
Hypertension	14.43	11.05	0.19	−0.51	0.83	0.54	−0.50	0.26	0.06
Depression	11.44	14.28	0.42	0.95	1.09	0.38	0.05	0.34	0.88

^a^Linear mixed effects model; age used as the time variable; O3PUFA at baseline includes plasma EPA+DHA; APOE4, apolipoprotein E epsilon 4 carrier; Gender, female; Education years; hypertension and depression require current treatment to qualify.

**Figure 1:**
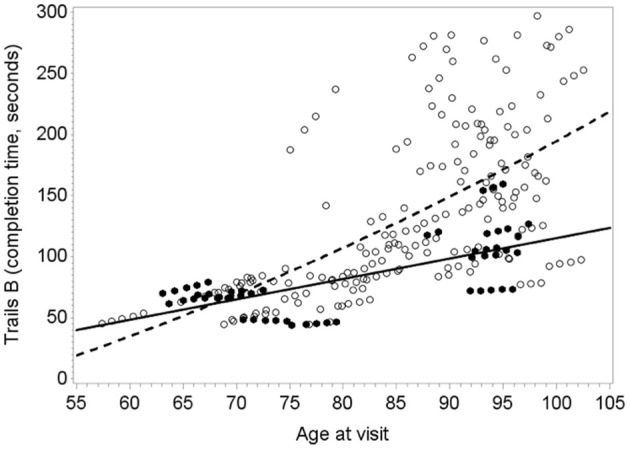
**Predicted within-person(circles) and mean trajectory (lines) of executive decline for subjects with plasma O3PUFA concentration above 110 μ g/ml (dark circles, solidline) or below this threshold(open circles, dashed line) at base line (*n* = 86).** Coefficient estimates calculated in the linear mixed effects model were used to predict trajectories. Participants with O3PUFA ≤110 μg/ml (open circles and line) had an accelerated rate of executive decline by 2.7 s per year (β = 2.7; 95% confidence interval −5.11 to −0.22; age, gender, education, *APOE4*, hyper tension, depression adjusted).

Figure 1 illustrates the deceleration in trajectory of executive decline in people with “high” plasma O3PUFA versus people below this threshold.

### Plasma O3PUFA and WMH mediated executive function (figure 2)

**Figure 2:**
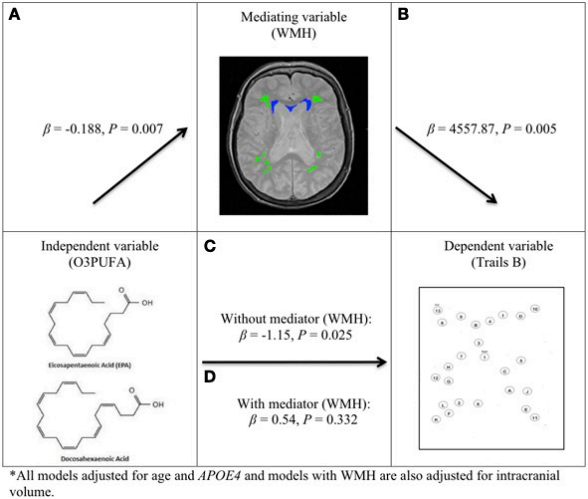
**O3PUFA and white matter mediated executive function in older adults (*n* = 32).** All models adjusted for age and *APOE4*, and models with WMH also adjusted for total intracranial volume. **(A)** Higher O3PUFA and less white matter hyper-intensities (WMH) highlighted in green (subcortical deep) and blue (periventricular). **(B)** Higher WMH and worse executive function (prolonged completion time for Trail B test). **(C)** Higher O3PUFA and better executive function (shorter completion time for Trail B test). **(D)** Association between O3PUFA and better executive function is lost once WMH is added to the regression model (*P* = 0.332) representing mediation.

**Figure 3:**
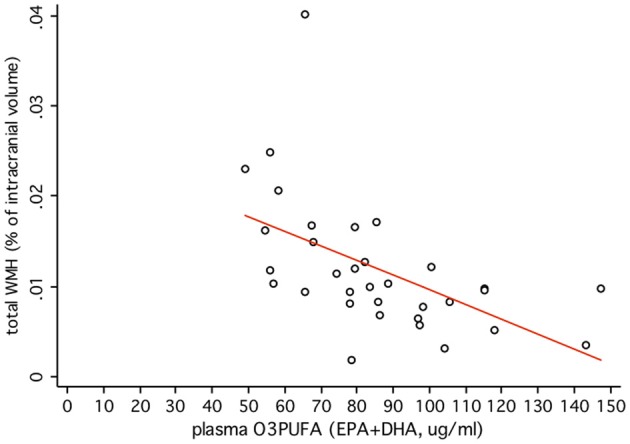
**Plasma O3PUFA explains 28.5% of the total variance in WMH in non-demented older adults (*n* = 32)**.

O3PUFA associated with less WMH (β = −0.188, *p* = 0.007, Figure 2A) where O3PUFA explained 28.5% of the variance in total WMH (Figure 3). WMH burden associated with worse executive function (Trials B, β = 4557.87, *p* = 0.005, Figure 2B). O3PUFA associated with better executive function (Tails B, β = −1.15, *p* = 0.025, Figure 2C), however, after adding WMH as a “mediating” variable into the regression equation that included O3PUFA, age, *APOE4* and total intracranial volume simultaneously as predictors of executive function, the significant association between O3PUFA and better executive function was lost (β = 0.54, *p* = 0.332, Figure 2D) and the WMH association with executive dysfunction remained marginally significant (β = 3589.95, *p* = 0.056, not illustrated in the Figure).

## Discussion

This longitudinal study of older adults at risk for dementia and followed over 4 years found significantly less executive decline in those with higher plasma O3PUFA at baseline. The calculated estimate indicates a 1-year delay in age-dependent executive decline per 100 μg/ml increase in plasma O3PUFA at baseline. We also found that plasma O3PUFA above 110 μg/ml associated with more stable executive function over time, which proposes one attainable threshold for neuroprotection. O3PUFA was not associated with verbal memory and MMSE. This implies that O3PUFA effects are more isolated to skills of executive function early on in people at risk for dementia. The mediation analysis further supports this notion since WMH accumulation is known to impact executive function and we demonstrate WMH mediation of the relationship between O3PUFA and executive dysfunction. Together, this evidence underlines important structural and functional brain parameters that seem well suited for targeting with O3PUFA therapy.

Delayed and logical verbal memory and MMSE changes were not associated with O3PUFA. However, executive decline appeared sensitive to O3PUFA. The mediation analysis is consistent with other literature indicating that prefrontal cortical executive skills are affected early during WMH accumulation (Schmidt et al., 1993; DeCarli et al., 1995; Adak et al., 2004; Brickman et al., 2006; Verdelho et al., 2007; Barbey et al., 2012). O3PUFA has pleiotropic effects that might explain this relationship, including effects on cerebral blood flow (Jackson et al., 2012), endothelial cell health (Yang et al., 2012), structural integrity of myelin (Pu et al., 2013), and preservation of neuronal energy with aging (Kuczynski et al., 2010).

Two other epidemiological studies have examined the relationship between O3PUFA and cognitive decline. Beydoun et al. (2007) were also unable to appreciate a relationship with global cognitive decline in the Atherosclerosis Risk in Communities Study. However, they did find O3PUFA associated with less decline in verbal/categorical fluency tasks which reflect executive control; requiring people to organize concepts in a novel way (i.e., naming words beginning with a particular letter or category with time constraints). They also report a more robust relationship in people with hypertension and dyslipidemia, two morbidities also sensitive to O3PUFA (Mozaffarian and Wu, 2011). Samieri et al. (2011) did not observe a relationship between O3PUFA (DHA or EPA) and MMSE change over 7 years in a Bordeaux subset of the 3 City study or an association with executive decline represented by Trails B. This inconsistency with our results in OBAS may be attributed to several factors, including differences in O3PUFA measures themselves (EPA and DHA as a relative percentage of total fatty acids versus an absolute concentration that appears more informative in our OBAS sample that has both), the age of the cohort (mean age 74 vs. 86 in OBAS), slower annual rates of executive decline (about 1 s/year decline in Trails B vs. 4 s/year in OBAS), and the potential for lower WMH prevalence in the Bordeaux sample compared with OBAS.

Several clinical trials to prevent age-related cognitive decline in the cognitively intact using O3PUFA therapy have been completed (van de Rest et al., 2008; Dangour et al., 2010; Geleijnse et al., 2012) and others are underway (Danthiir et al., 2011). Each completed trial has been unsuccessful, however, the current study results suggest that the design of the clinical trials themselves may explain the null effect. For example, one trial enrolled non-demented subjects age 66–74 and followed subjects for only 6 months and executive function was examined (van de Rest et al., 2008). Another trial included secondary outcomes of executive function (e.g., digit span backwards and animal naming), but Trails B itself was not administered, and the population was at low vascular risk, younger age, and follow-up duration was limited at 2 years (Dangour et al., 2010). Geleijnse et al trial did examine a population with vascular risk (Geleijnse et al., 2012), but employed the MMSE as the primary outcome, which now has consistently shown to be insensitive to O3PUFA in observational and experimental studies of older adults at risk for dementia.

There were limitations in our study. We have plasma O3PUFA measurements available from a single time point in each participant, and we assume that this is a reasonable representation of long-term O3PUFA status, the type of nutritional exposure most inherently significant to brain aging. Our sample size is smaller than studies of cognitive aging and incident dementia using subjective measures of dietary intake (i.e., food frequency questionnaire). However, studies using self-report are susceptible to measurement error that we circumvent by utilizing quantitative nutrient biomarkers (Bowman et al., 2011; Tangney and Scarmeas, 2012). These permits more power, and, in turn a more conservative sample size to identify important relationships.

In conclusion, these results add longitudinal data to a limited body of literature that further indicate WMH and executive function as features of cognitive aging that appear sensitive to O3PUFA early in the evolution of cognitive decline. The hypothesis that O3PUFA can prevent vascular cognitive aging warrants further study.

## Conflict of interest statement

The authors declare that the research was conducted in the absence of any commercial or financial relationships that could be construed as a potential conflict of interest.
