# Supplementary material for: Plasma omega-3 PUFA and white matter mediated executive decline in older adults
Source: Front Aging Neurosci. 2013 Dec 16;5:92. doi: 10.3389/fnagi.2013.00092 (PMC3863786; doi:10.3389/fnagi.2013.00092)
Supplement: Supplementary file 1 [file DataSheet1.PDF]

***e*Table 1. Demographic and clinical characteristics associated with Trail Making Test Part B in non-demented elders with MRI derived WMH available using linear regression (n=32)**

	β	**SE**	***P***	**95% CI**
**Age**	9.65	3.78	0.017	1.87-17.42
**Gender**	11.14	31.32	0.725	-53.24-75.52
**Education**	-1.27	5.89	0.831	-13.37-10.83
***APOE4* carrier**	161.05	75.38	0.042	6.10-315.99
**Hypertension**	22.66	25.74	0.387	-30.25-75.56
**Depression**	44.15	35.44	0.244	-28.69-116.99
